# Research on Minimization of Data Set for State of Charge Prediction

**DOI:** 10.3390/s22031101

**Published:** 2022-01-31

**Authors:** Tun Liu, Jundong Zhao, Chaoqun Xiang, Shu Cheng

**Affiliations:** School of Traffic & Transportation Engineering, Central South University, Changsha 410075, China; liutuncsu@csu.edu.cn (T.L.); zhaojd@csu.edu.cn (J.Z.); xiangchq@csu.edu.cn (C.X.)

**Keywords:** long short-term memory (LSTM), state of charge, principal components analysis (PCA), data fusion

## Abstract

The quick estimation and prediction of lithium-ion batteries’ (LIBs) state of charge (SoC) are attracting growing attention, since the LIB has become one of the most essential power sources for daily consumer electronics. Most deep learning methods require plenty of data and more than two LIB parameters to train the model for predicting SoC. In this paper, a single-parameter SoC prediction based on deep learning is realized by cleaning the data for lithium-ion battery parameters and constructing the feature matrix based on the cleaned data. Then, by analyzing the feature matrix’s periodicity and principal component to obtain two kinds of the original eigenmatrix’s substitution matrices, the two substitutions are fused to obtain an excellent prediction effect. In the end, the minimization method is verified with newly measured lithium battery data, and the results show that the MAPE of the SoC prediction reaches 0.96%, the input data are reduced by 93.33%, and the training time is reduced by 96.68%. Fast and accurate prediction of the SoC is achieved by using only a minimum amount of voltage data.

## 1. Introduction

The lithium-ion battery (LIB) has become one of the essential mobile power sources, from electric vehicles (EVs) to cell phones and from microgrids to laptops, owing to its mature technology, low cost, high energy density, and long service time [1]. It is more and more critical to quickly and accurately obtain an LIB’s state of charge (SoC). However, most of the SoC estimation methods proposed in the literature are based on studies of large equipment that are conducted during the service process, such as with vehicles. This paper investigates LIB’s initial SoC in daily consumer electronics to provide the convenience of future edge computing and the Internet of Things. The initial SoC is helpful to estimate the SoC in use accurately, and is an essential indicator of the state of health (SoH). This paper aims to quickly and accurately predict LIB’s SoC with minimum data.

The existing SoC estimation and prediction methods can be divided into two categories: direct methods and indirect methods. The direct methods include electrochemical impedance spectroscopy (EIS), current integral [2], and open-circuit voltage (OCV) methods, which are dependent on the battery’s measurable variables [3]. The indirect methods usually exploit data-driven methods and model-based methods. The data-driven approaches can be classified into the observer method [4], variants of the Kalman filter (KF) [5,6,7,8], the particular filter (PF) [9], and neural network-based deep learning methods [10]. Some data-driven methods also depend on the battery models, which are classified into electrochemical and circuit models.

EIS needs a specific measuring instrument to obtain ohmic resistance, charge resistance, and noise sensitivity, which cannot be adopted for online estimation [11,12,13]. Current integral/coulomb-counting estimation needs to know the SoC, and relies significantly on precise current measure sensors. Additionally, the EIS is easily affected by temperature variations, the C-rate, and accumulated errors [14,15]. OCV must have a predefined table describing the monotonic relationship between OCV and SoC [16], and needs rest time to obtain the precise value.

Various model-based SoC estimation approaches have been proposed. The electrochemical mechanism model [17] can describe the inherent battery mechanisms, but it is complicated and time-consuming. The equivalent circuit model (ECM) usually uses first-order or second-order models that utilize parallel RC elements (open-circuit voltage terms and resistances) to model the battery’s dynamic behavior [18], and is combined with a data-driven classification algorithm. The first-order models are widely adopted by unscented Kalman filter (UKF) [6], extended Kalman filter (EKF) [7], double particle filter (DPF) [8], sliding-mode observer (SMO) [19], discrete-time nonlinear observer (DNLO) [4], and dual H infinity filter [20] models. There are some second-order models applied in EKF [21] and support vector machine (SVM) [22] models. Other methods, such as mathematic models, use the improved Volterra equations [23] to dynamically describe the battery’s degradation. Though these methods achieve satisfactory performance, building a precise model is still challenging, and plenty of experimental data are necessary.

Currently, neural network-based deep learning methods are the state-of-the-art methods for various applications. Deep learning is model-free and usually only dependent on the battery’s measurable variables. The typical deep learning algorithms include long short-term memory (LSTM) [24,25], convolutional neural networks (CNN) [26,27,28,29], and complex CNN-LSTM [30,31]. The deep learning methods can save the tedious work of building the model, but they need a large amount of data from more than two parameters.

This paper proposes a novel approach combined with LSTM to realize promising SoC prediction with only voltage data. The principal component analysis and periodic analysis obtained the feature matrix’s substitution components, and then we fused the two parts. Combining these two kinds of data can enlarge the difference between the original matrices and achieve a better prediction effect. Simultaneously, this method reduces the amount of input data by more than 93% and reduces the training time by 96%.

## 2. Construction of Data Set

### 2.1. Data Background

**Figure 1:**
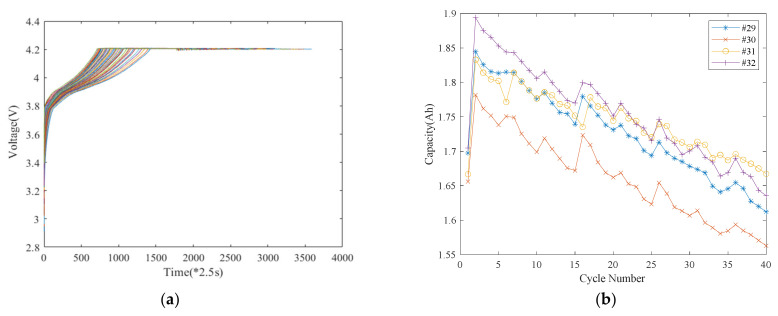
(**a**) CC-CV charging curve; (**b**) discharging capacity.

The NASA lithium-ion battery data sets from the Prognostics Center of Excellence Data Repository [32] are chosen for the experiment that only uses voltage data for SoC prediction. The data were collected under the constant current–constant voltage (CC-CV) principle at 43 °C. Specifically, the charging was conducted at a constant current of 1.5 A until the battery reached the upper charging limit of 4.2 V, and then it continued in constant voltage (CV) mode until it reached the current cut-off at 20 mA. The discharging was conducted under a constant current of 4 A until the cell voltage dropped to 2.0 V, 2.2 V, 2.5 V, and 2.7 V (Table 1). The LIB voltage curves are shown in Figure 1a, and the capacity curves are shown in Figure 1b. This paper uses the #30, #31, and #32 batteries’ charging voltage data as training data and the #29 battery’s data as test data.

### 2.2. Characteristic Parameter Cleaning

Selecting appropriate test parameters can reduce the required parameter types and the amount of test data. The measurable data of the battery are mainly pressure, temperature, internal resistance, voltage, and current. The characteristics of these parameters will be analyzed next.

1. Pressure

A.J. Louli [33] found that the irreversible capacity loss of batteries was correlated with the growth of SEI film by studying the negative electrode of different batteries, including the NCA of a ternary lithium battery, the LiCoO2 of a cobaltate lithium battery, and the NMC of a nickel manganese cobalt battery, and discovered that the thickening of the SEI film could be measured by the pressure. During charging, the overall pressure of the battery increases because of the lithium embedded in the negative electrode, which increases the volume of the battery. Conversely, the pressure drops during discharge. The tension generated on the SEI film is weak, and high-precision pressure sensors are often required. In A.J. Louli’s study, the sensor used for measurement was the ultra-small industrial compression weighing sensor Omega-LCKD, and the sensor data processor is OMEGA dp25B-S. However, the sensor and data processor are very high precision and expensive, suitable only for the laboratory.

2. Temperature

**Figure 2:**
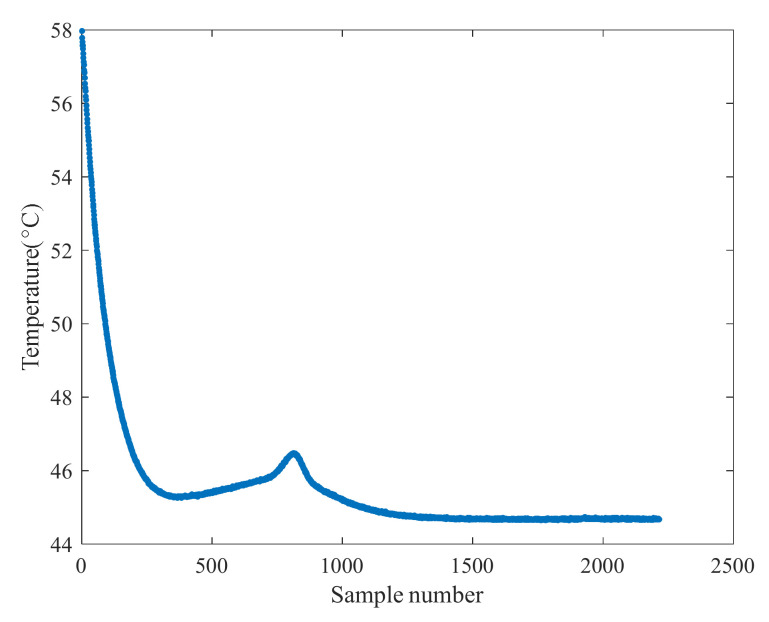
Battery temperature change curve.

Battery heating is mainly concentrated in the battery interior, and even the internal heating is uneven due to the influence of the manufacturing process. It can be seen from the curve of NASA’s single temperature measurement in Figure 2 that the temperature varies over a wide range and takes a long time to stabilize, which also indicates that it is difficult to accurately obtain the internal temperature of the battery through external measurement. It is not an appropriate single parameter to characterize battery degradation. Simultaneously, the external environment easily affects the temperature and pressure parameters.

3. Internal resistance

**Figure 3:**
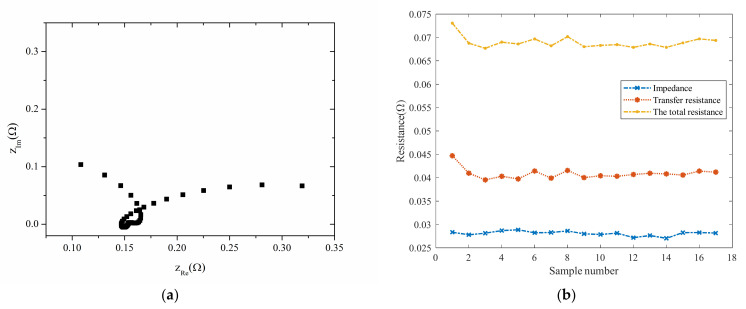
Internal resistance: (**a**) impedance Nyquist diagram; (**b**) resistance.

The internal resistance of lithium-ion batteries is usually measured using electrochemical impedance spectroscopy (EIS). An equivalent circuit usually represents the internal resistance of each part of a battery. The resistance values of each part of the equivalent circuit can be obtained by combining the equivalent model with the analysis of the Nyquist diagram. However, the process is complicated by placing the battery in a particular instrument to test it. The battery’s impedance obtained from the NASA data can be seen in Figure 3a. It is not easy to intuitively obtain the impedance, which requires particular analysis software or an AC impedance spectrum tester to analyze. Meanwhile, it can be seen from Figure 3b that the total resistance change of the whole cycle test does not reflect capacity degradation.

4. Voltage and current

Generally, battery management systems provide voltage and current detection functions that do not need additional sensors. However, the current parameter is affected by environmental noise, the load, and the control of the charging instrument. Voltage is the external reflection of the migration of elements inside the battery, and it is not affected by the external environment, making it an ideal parameter for characterizing the degradation of energy storage devices [34,35]. When charged at a certain current, the voltage can reflect the physical and chemical characteristics of the battery better than the current.

### 2.3. Data Set Construction

**Figure 4:**
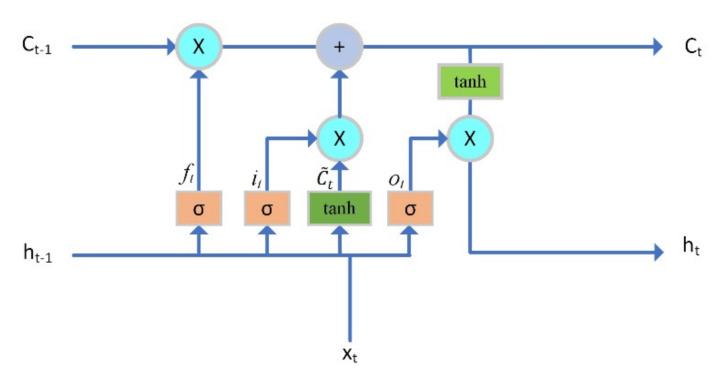
LSTM cell structure.

The LSTM network can store long-term and short-term states in two cells, *C_t_* and *h_t_*, respectively (Figure 4). $C_{t}$ denotes the memory cell state, which can store long-term data features. $h_{t}$ stores short-term states. The fundamental of LSTM is that the state of the memory cell *C_t_* can be well propagated and store data features from a long time ago [24]. Therefore, The LSTM deep learning algorithm has the advantage of processing sequence data, and the voltage data are precisely this kind of data. The definitions of the forget gate $f_{t}$, input gate $i_{t}$, candidate state ${\tilde{C}}_{t}$, new cell state $C_{t}$, output gate $O_{t}$, and hidden state $h_{t}$ are calculated as follows (1)–(6):

(1) $$f_{t}=\sigma \left(w_{f}\cdot \left[x_{t},\;\;h_{t-1}\right]+b_{f}\right)$$

(2) $$i_{t}=\sigma \left(w_{i}\cdot \left[x_{t}\;,\;h_{t-1}\right]+b_{i}\right)$$

(3) $${\tilde{C}}_{t}=\tan h\left(w_{c}\left[x_{t},\;h_{t-1}\right]+b_{c}\right)$$

(4) $$C_{t}=f_{t}ʘC_{t-1}+i_{t}ʘ{\tilde{C}}_{t}$$

(5) $$O_{t}=\sigma \left(w_{O}\cdot \left[x_{t},\;h_{t-1}\right]+b_{O}\right)$$

(6) $$h_{t}=O_{t}ʘ\tan h\left(C_{t}\right)$$

**Figure 5:**
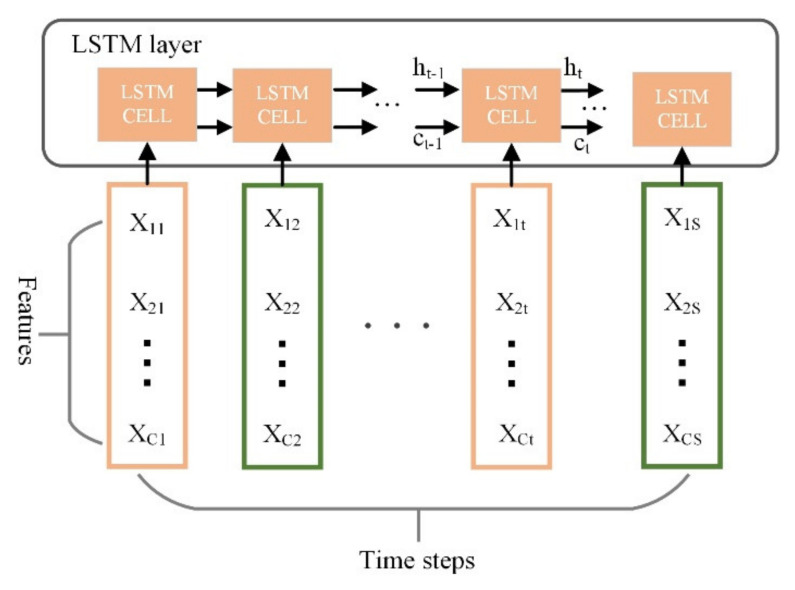
LSTM work schematic diagram.

The LSTM algorithm uses a column vector as an input feature (Figure 5). The voltage data of each cycle are converted into a 40 × 40 matrix so that the deep learning models can process it. According to the characteristics of the LSTM, every 40-interval data point of a column is taken as two adjacent factors to make sure that the matrix has the most significant standard deviation in the column. 

Then, the voltage data and voltage derivative are combined column by column after being normalized by the z-score method. The normalizations of xmin-xmax, atan, and log limit the output value in the ranges of 0 to 1, −1 to 1, and 0 to 1, respectively, which cannot increase the difference between the data. Therefore, the data are normalized with the z-score method, which places no restrictions on the mapping range of the data, as shown in Equations (7)–(9):

(7) $$\mu =\frac{1}{n}\sum_{i=1}^{n}x_{i}$$

(8) $$s=\sqrt{\frac{1}{n-1}\sum_{i=1}^{n}{\left(x_{i}-\mu \right)}^{2}}$$

(9) $${\tilde{x}}_{i}=\frac{x_{i}-\mu }{s}\;\;\;\;\;\;\;i=1,\;2,\cdots ,n$$

where μ is the mean value, s is the standard deviation, and ${\tilde{x}}_{i}$ is the normalized value.

**Figure 6:**
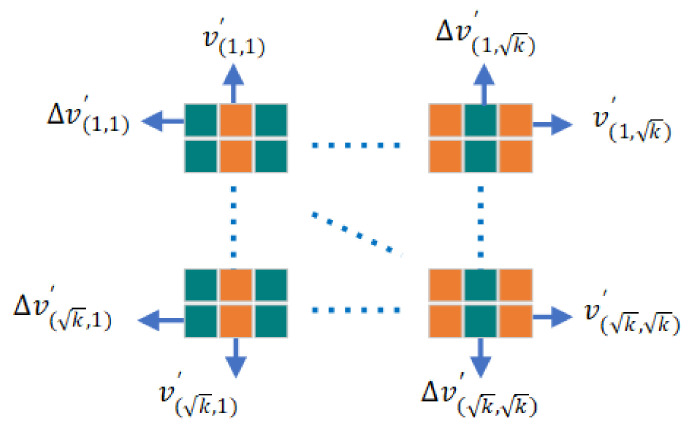
The data arrangement. Where $v^{\prime }$ is the normalized voltage value and $\Delta v^{\prime }$ is the normalized voltage derivative value.

**Figure 7:**
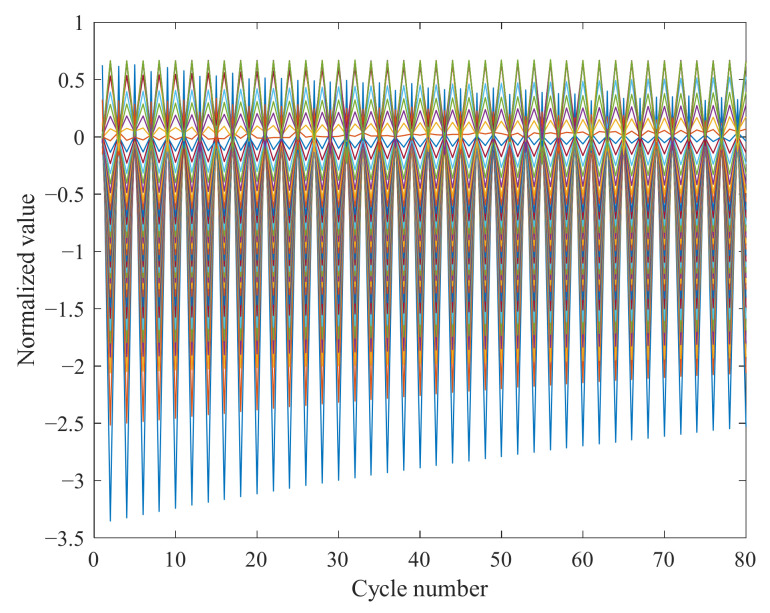
The data matrix visualization.

The voltage derivative is imported as input data to enlarge the feature of the data set, then arranged with voltage data according to the characteristics of the LSTM algorithm, the arrangement method of which is illustrated in Figure 6. Figure 7 shows the visualization of the arrayed matrix.

## 3. Analysis of Critical Features of Data Set

The constructed matrix uses up to 3200 pieces of data, and a total of 512,000 pieces of data are used throughout the test cycle of the four batteries. If the battery has more cycles, the amount of data used will be larger, which will increase the calculation burden and training time, so the data need to be minimized.

### 3.1. Data Periodicity Analysis

One way to reduce the dimensionality of the data is to replace the original data with its minimum period data if the data are periodic. From the visualization of NASA’s battery data matrix in the previous section, the amplitude varies roughly periodically. In order to verify its periodic change, the correlation index between the even and odd columns of the matrix is analyzed by using the correlation coefficient function in Equation (4). For even columns, take the first two columns 1–2, 1–4, 1–6, and 1–8 with the subsequent even sequence, such as (i + 1)–(i + 2) column i = 2,4,6,…, k/2; (i +1)–(i + 4) column i = 4,6, …, k/4; (i +1)–(i + 6) column i = 6, …, k/6; (i + 1)–(i + 8) column i = 8, …, k/8, and carry out the correlation coefficient analysis. The odd column is the first 1–3, 1–5, 1–7, and 1–9 and the following odd sequence, such as (i + 1)–(i + 3) i = 3, 5, 7, …, k/3; (i + 1)–(i + 5) column i = 5,7, …, k/5; (i + 1)–(i + 7) column i = 7, …, k/7; (i + 1)–(i + 9) column i = 9, …, k/9. Thus, the correlation coefficient analysis was performed. The formula for the correlation coefficient is as follows:

(10) $$Corr=\frac{\sum_{m}\sum_{n}\left(A_{mn}-\bar{A}\right)\left(B_{mn}-\bar{B}\right)}{\sqrt{\sum_{m}\sum_{n}{\left(A_{mn}-\bar{A}\right)}^{2}}\sqrt{\sum_{m}\sum_{n}{\left(B_{mn}-\bar{B}\right)}^{2}}}$$

**Figure 8:**
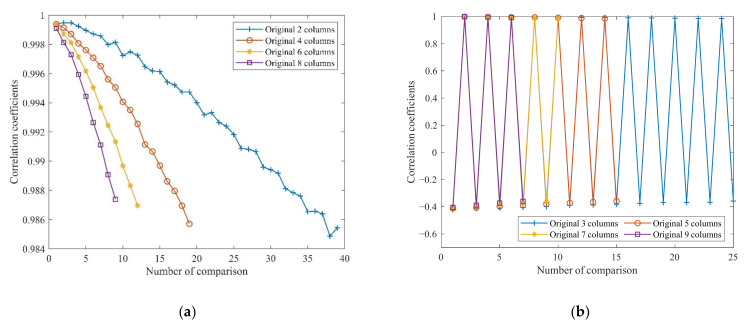
The correlation coefficients of the matrix of (**a**) even columns and (**b**) odd columns.

Figure 8a shows that the correlation coefficient between even columns presents an approximate linear downward trend, but the correlation coefficients remain above 0.98. The high correlation coefficients indicate a high similarity and linear relationship between even columns. The correlation coefficients of odd columns in Figure 8b show significant fluctuation and poor similarity, so odd columns cannot be used to replace the remaining data.

We changed the discrete sampling signal into the time domain signal with the sampling period of 1 s steps, then analyzed its periodicity through the Fourier transform equation in Equation (11):

(11) $$x_{k}=\overset{N-1}{\underset{n=0}{\sum }}x_{n}e^{-j\frac{2\pi }{N}k*n}\;\;\;\;\;\;\;\;\;\left(k=0,1,2\cdots N-1\right)$$

The Euler expansion of the above formula can be obtained in (12):

(12) $$x_{k}=\overset{N-1}{\underset{n=0}{\sum }}x_{n}(cos\left(2\pi k\frac{n}{N}\right)-jsin\left(2\pi k\frac{n}{N}\right)\;\;\;\;\;\;\;\;$$

**Figure 9:**
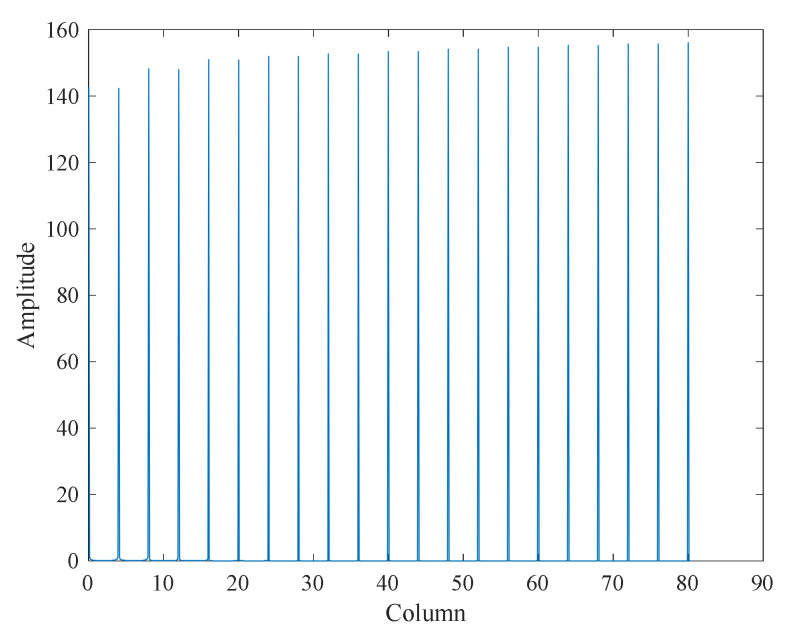
Fourier analysis diagram.

After Fourier series expansion, the spectrum is converted to the ordinate and listed as columns. It can be seen that the amplitude of the discrete time domain signal also changes periodically every four columns, as shown in Figure 9.

### 3.2. Data Difference Analysis

**Figure 10:**
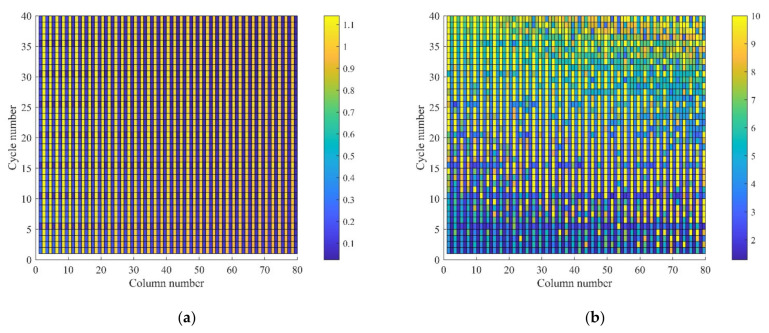
The global diagram for (**a**) standard deviation and (**b**) ACV.

The global standard deviation (Figure 10a) shows that although there are periodic changes, the standard deviation difference between an odd column and even column is too large. The difference in each column is too small to distinguish the data that can provide more different features.

The absolute coefficient of variation (ACV) (Equation (13)) is introduced in this section to further analyze the degree of data dispersion. The absolute value of the ratio of the standard deviation to the mean is the absolute coefficient of variation, which can be used to measure the relative discrete relationships between data. The smaller the coefficient of variation is, the smaller the dispersion degree of the data is; the larger the coefficient of variation is, the greater the dispersion degree of the data is.

(13) $$\mathrm{ACV}=\left|\frac{s}{\mu }\right|$$

**Figure 11:**
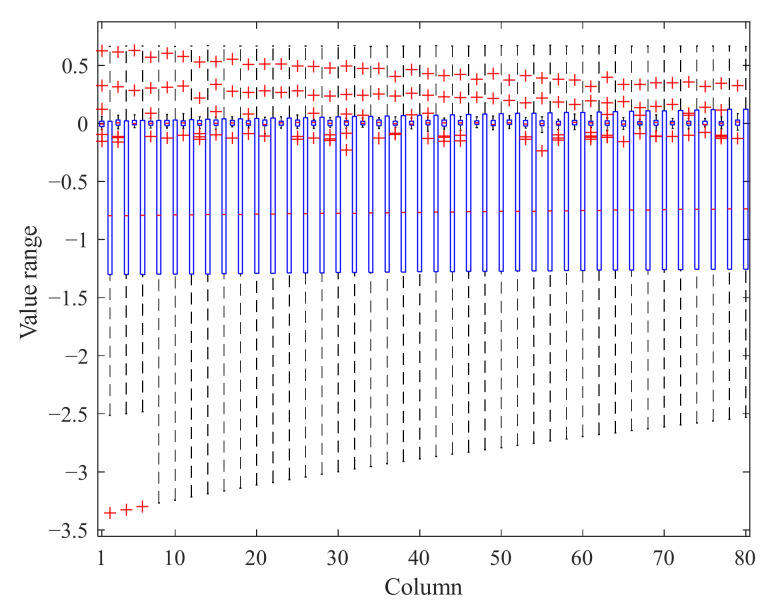
Box plot of a single cycle.

The global diagram of ACV is generated with the all circulating data of NASA in line with the column direction, as shown in Figure 11. In the graph, the abscissa is the number of columns of the matrix, and the ordinate is the number of cycles. To better analyze the ACV distribution, the maximum ACV is limited to 10. Figure 10b shows that the column numbers with a large coefficient of variation are evenly distributed.

The global analysis results cannot provide data differences within a single cycle. Therefore, the box plot in statistics is introduced to analyze data variability within a single cycle. In the box plot of the NASA data (Figure 11), it can be seen that in the even columns, the median of even columns is basically the same, and the quartile distance is not significantly different, while the median of the odd number columns fluctuates slightly. The variation decreased with the increase in the column number. Thus, we consider taking the even columns in front of the matrix as a substitute for the whole matrix.

### 3.3. Principal Component Analysis

Principal component analysis (PCA) is another widely used data feature substitution method (1–3) adopted to find the matrix variation’s principal components. Finding the principal components to replace the original matrix through PCA can reduce the size of the original matrix and the amount of data.

The steps of PCA are as follows:

1. Normalization of the matrix

Establish the input matrix $X={\left[x_{1},x_{2},\cdots ,x_{m}\right]}^{T}$ (14), where $x_{i}=({\tilde{x}}_{i1},{\tilde{x}}_{i2},\cdots ,{\tilde{x}}_{in}$). ${\tilde{x}}_{ij}$ is computed by Equation (15):

(14) $$X=\left[\begin{array}{ccc} x_{11} & \cdots & x_{1n}\\ \vdots & \ddots & \vdots \\ x_{m1} & \cdots & x_{mn} \end{array}\right]$$

(15) $${\tilde{x}}_{ij}=\frac{x_{ij}-\mu }{s}\;(i=1,2,\cdots ,m;j=1,2\cdots ,n)$$

where $\mu =\frac{1}{n}\overset{n}{\underset{i=1}{\sum }}x_{i}$ and $s=\sqrt{\frac{1}{n-1}\overset{n}{\underset{i=1}{\sum }}{\left(x_{i}-\mu \right)}^{2}}$

2. Correlation coefficient calculation

The normalized data are used to calculate the correlation coefficient matrix C = (r_ij_)m×n (16), where $c_{ii}$ = 1 and $c_{ij}=c_{ji}$. $c_{ij}$ denotes the correlation coefficient between the *i*th column and the *j*th column (17).

(16) $$C=\left[\begin{array}{ccc} r_{11} & \cdots & r_{1n}\\ \vdots & \ddots & \vdots \\ r_{m1} & \cdots & r_{mn} \end{array}\right]$$

(17) $$c_{ij}=\frac{\sum_{k=1}^{m}{\tilde{a}}_{ki}\cdot {\tilde{a}}_{kj}}{m-1}\;(i,\;j=1,2,\cdots ,m;k<i,j\;)$$

where ${\tilde{a}}_{ki}=\left(x_{ki}-{\tilde{x}}_{ki}\right)$ and ${\tilde{a}}_{kj}=\left(x_{kj}-{\tilde{x}}_{kj}\right)$.

3. Eigenvalue and principal matrix

(18) $$\left|\lambda E-C\right|=0$$

Through Equation (18), the eigenvalue of *R* can be obtained, where $\lambda =(\lambda_{1},\lambda_{2}\cdots \lambda_{m}$) and eigenvector $u={\left(u_{11},u_{2j,}\cdots ,u_{mj}\right)}^{T}$. $y_{i}$ denotes the ith principal component of the data matrix. The principal matrix $Y=\left[y_{1},y_{2},\cdots ,y_{m}\right]$ is shown in (19):

(19) $$\left[y_{1},y_{2},\cdots ,y_{m}\right]=\left[\begin{array}{ccc} x_{11} & \cdots & x_{1n}\\ \vdots & \ddots & \vdots \\ x_{m1} & \cdots & x_{mn} \end{array}\right]\left[\begin{array}{ccc} u_{11} & \cdots & u_{1n}\\ \vdots & \ddots & \vdots \\ u_{m1} & \cdots & u_{mn} \end{array}\right]$$

4. Contribution ratio

**Figure 12:**
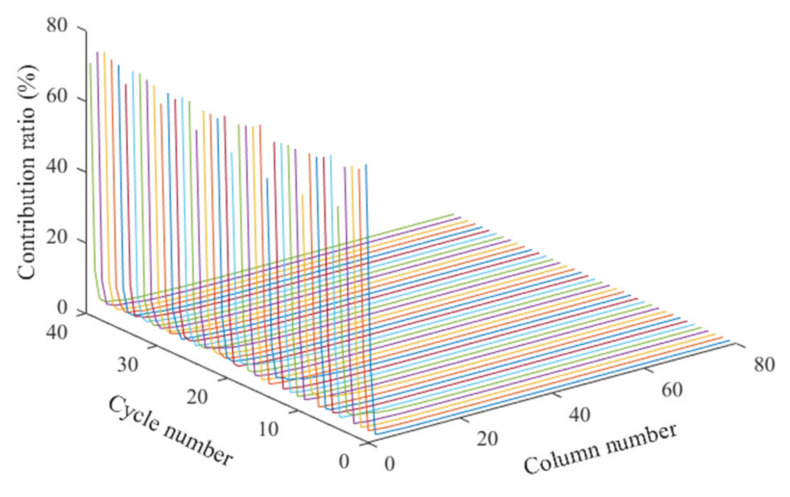
The contribution rate of PC matrices.

The contribution $b_{j}$ of $y_{j}$ is calculated through Equation (20), and ap in Equation (21) is the accumulative contribution rate:

(20) $$b_{j}=\frac{\lambda_{j}}{\sum_{k=1}^{m}\lambda_{k}}\;$$

(21) $$a_{p}=\frac{\sum_{k=1}^{p}\lambda_{k}}{\sum_{k=1}^{m}\lambda_{k}}$$

when *a_p_* is close to 1 (>85%) and the *p* PC matrix is usually chosen to replace the original matrix. Figure 12 shows that the contribution rate of the first several columns in the principal component matrix is the largest, and the contribution rate of the subsequent columns decreases sharply. The accumulative contributions of PC8 and PC20, respectively, are over 88% and 98%.

## 4. Prediction and Minimization

### 4.1. Prediction Based on Periodic and Principal Components Data

In this section, the even-numbered columns of the original matrix and the principal component matrix are respectively tested by the LSTM. The testing platform is equipped with an Intel Core (TM) i7-9750H CPU, 16 GB RAM, and NVIDIA GeForce GTX 1650 Max-Q GPU. The LSTM used has five layers: the first layer is the sequence layer, which is set to 30 according to the matrix characteristics parameter, and the second layer is the LSTM functional layer; the third layer is the full connection layer; the fourth layer is the normalized function layer; the fifth layer is the classification layer. Training success is defined by a training accuracy of over 98%. The SoC is defined in Equation (10), where $Q_{\mathrm{rated}}$ is the battery’s rated energy and $Q_{\mathrm{remain}}$ is the battery’s remaining energy. When the Qremain is the next maximum releasable energy after the battery is fully charged, the initial SoC can be obtained (22). The mean squared error (MSE) (23) and the mean absolute percent error (MAPE) (24) are used as error evaluation indicators.

(22) $$\mathrm{SoC}=\frac{Q_{\mathrm{remain}}}{Q_{\mathrm{rated}}}$$

(23) $$MSE=\frac{1}{k}\sum_{k=1}^{k}{\left({\tilde{C}}_{k}-C_{k}\right)}^{2}$$

(24) $$MAPE=\frac{1}{k}\sum_{k=1}^{k}\left|\frac{{\tilde{C}}_{k}-C_{k}}{C_{k}}\right|*100\%$$

**Figure 13:**
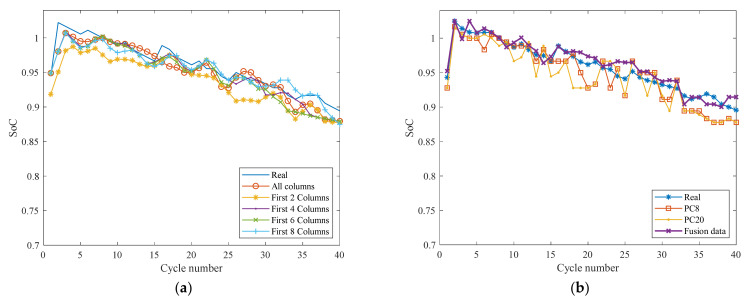
The prediction result of the (**a**) original matrix with different columns and (**b**) PC matrices.

The prediction results are shown in Figure 13 and Table 2. Table 2 illustrates that when the complete normalized matrices are used, the MSE and MAPE are 2.56 × 10^−4^ and 1.42%, respectively. Though a good prediction was obtained in the test, the model was trained by using a large amount of input data in one minute and thirty-eight seconds. A large amount of input data increases the computational load and the data sampling and storage costs. Additionally, it delays the results output. This is challenging for online real-time prediction. When using different even columns for prediction, the training time and input data are significantly reduced, but the prediction effect is slightly worse than the original matrices.

Overall, the prediction results of using 1–4 original columns are slightly worse than those using the complete original matrix. In contrast, using 1–4 original columns can reduce the training time and the data use ratio by 96% and 95%, respectively. Nevertheless, the prediction effect of the principal component matrices is much worse than that of the original matrices.

### 4.2. Minimization

The prediction results of periodic data substitution and principal component substitution are worse than those of the original matrix, but significantly reduced the training time. Since both data substitutions represent the main features of the original matrix, further fusion is considered for testing the prediction effect. To combine the advantages of both substitution matrices, we created a matrix by combining the principal components with the periodic data, as shown in (25):

(25) $$X={\left[x_{1},x_{2},x_{3},x_{4},PC_{1},PC_{2},PC_{3},\cdots ,PC_{8}\right]}^{T}$$

In the first four columns of the matrix, $x_{1-4}$ is the first four columns of the original data, and the next eight columns are the eight principal components of $PC_{1-8}$. After standardization to calculate the correlation coefficient $C_{ij}$, the eigenvalue λ and eigenvectors U are then obtained by solving the eigenequation using the Jacobian matrix method. A new fusion matrix is obtained by multiplying the first four eigenvectors of the composite matrix X and the corresponding matrix U. The cumulative contribution rate of the new fusion data’s first four principal components is more than 92%. Figure 13b displays the prediction curve.

Table 2 shows that the MSE and MAPE of the fusion data are reduced by 0.46% and 30.47%, respectively, and the training time is also reduced by 96%. We conclude that the fusion data, combining the advantages of the two alternative data, has the best prediction effect.

#### Verification

We tested four LIBs (LIR2032) under the CC-CV charging process at room temperature to verify the previous analysis results. The charging was conducted under a constant current (CC) of 20 mA until it reached the upper voltage limit of 4.2 V. Then, it was continued in constant voltage (CV) mode until the charge current dropped to 2 mA. The discharging was conducted under a constant current of 20 mA until the voltage dropped to 3 V. In the discharging process, we obtain the available discharging capacities. Table 3 lists the work conditions.

The rated capacity of the new LIB was 35 mAh, which is lower than that of the NASA data, resulting in fewer sample numbers, but more new LIB test cycles. First, the testing data are normalized and changed to 30 × 60 matrices with the voltage data and voltage derivative. Then, we calculated the principal components of the four batteries’ charging data through PCA. Next, the predictions of SoC based on the matrix that takes the same columns from the original matrix via the LSTM model were compared. The #1–3 LIB charging voltage data are used as training data, and the #4 LIB charging voltage data are used as testing data. The amount of data for the test is 624,600, accounting for 58.28% of the total testing data of 1,071,722.

**Figure 14:**
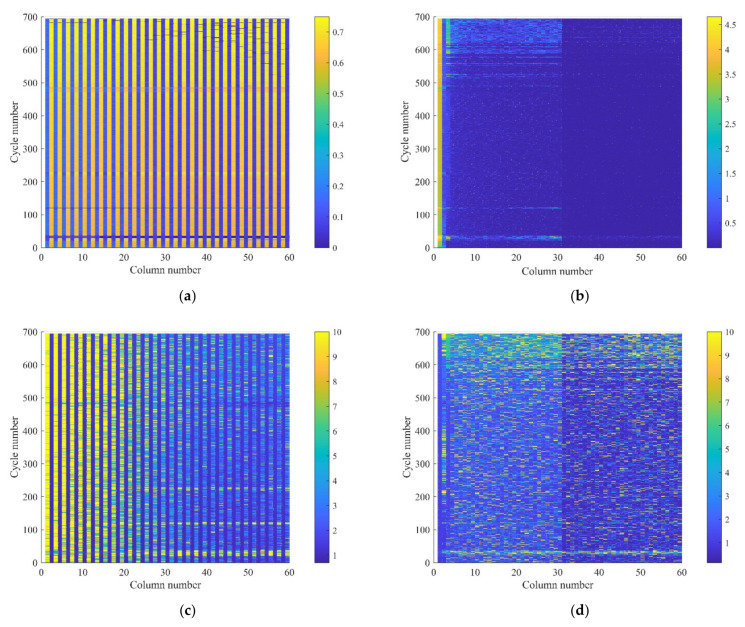
The (**a**) standard deviations of 694 cycles and (**b**) PC matrices; the ACV of (**c**) original matrices and (**d**) PC matrices.

We found that a few PCs still account for a large proportion of the contribution, and the accumulative contributions are over 88% and 98% when taking four PCs and 14 PCs, respectively. The new batteries’ matrices display periodic changes in every two columns, and the even columns have a correlation coefficient of over 0.96 with the subsequent even columns. The average standard deviation of even columns is much smaller than the change in PC, and fluctuates around 0.5. Figure 14a shows the standard deviation of the original matrix, which shows periodic changes in the even-numbered columns; in Figure 14b, it rapidly decreases as the number of columns increases. Besides, Figure 14c illustrates that the high ACV values appear in the first few columns; Figure 14d indicates that the ACV of the PC matrices are distributed evenly among the columns and cycles. Then, the periodic data and principal component data of the measured data are fused, and the three kinds of data are tested separately.

**Figure 15:**
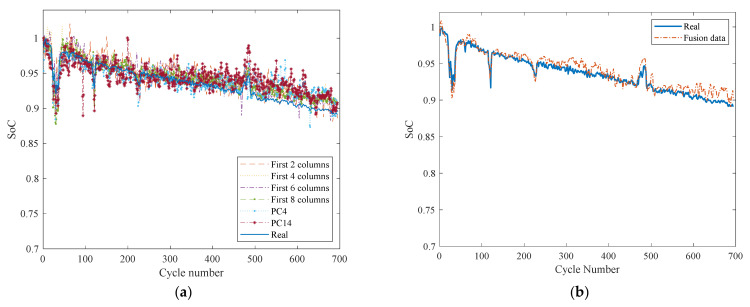
The prediction results of (**a**) a variety of PC matrices and the original matrix with different columns and (**b**) fusion data matrix.

Figure 15a and Table 4 show that the MSE and MAPE of using the original full matrices are 2.14 × 10^−4^ and 1.37%, respectively. Using two columns from the original matrix, the MSE and MAPE increased by 105.6% and 45.8%, respectively; using 1–4 columns of the original matrix, the MSE and MAPE respectively increased by 10.3% and 6.7%, and the training time and data used ratio respectively decreased by 92.9% and 95%. When using PC4 matrices, the MSE and MAPE increased by 69.6% and 22.5%, respectively; for PC14 matrices, the MSE and MAPE increased by 143% and 56.7%, respectively. The MSE and MAPE of 1–4 columns are 132.7% and 50% lower than PC14. This verifies that the prediction based on directly using the even columns of the matrices composed of voltage and voltage derivatives is better than that of the PC matrices. However, the training speed of using the principal component is much faster than using the even columns. The results listed in Table 4 demonstrate that the MSE of the first four columns and MAPE respectively increase only by 10.28% and 6.67% compared with the complete matrix and the PC4, with an accumulating contribution rate over 88%. Therefore, the study considers combining the even columns and PC4 to achieve their prediction accuracy and speed advantage.

Figure 15b shows that the prediction curve of the principal components combined with the first four columns exhibits promising results. Table 4 illustrates that the MSE and MAPE reach the lowest values of 2.09 × 10^−4^ and 0.96% in all predictions, which are 9.16% and 20.00% lower than the complete matrix, respectively. The input data were reduced by 93.33%, from the original 1800 to 120, accomplishing the goal of using the minimum amount of input data to achieve an accurate prediction of an LIB’s SoC. Meanwhile, the training time was reduced by 67.46% and 96.68%, compared with the original first four columns and the original matrix, respectively.

## 5. Conclusions

The normalized voltage data and voltage derivative data are combined to form a new matrix with a high similarity (the correlation coefficient value is over 0.96) between even-numbered columns, realizing SoC prediction based on a single parameter. We compared the predictions based on the new matrices and the PCA matrices, which consisted of PCs with a cumulative contribution rate of more than 85%, and found that the former predictions are better than those based on the PC matrices. The prediction effect of both alternate data is worse than that of using the original matrix, but the training time is much shorter.

Then, the prediction effect was tested by fusing data. The fusion matrix of PCs and even columns contribute to better prediction results than the complete matrix, with an MSE of 2.09 × 10^−4^ and a MAPE of 0.96%. By minimizing the data set, we achieved high-precision SoC prediction with less than 10% of the input data based on a single parameter. Simultaneously, the novel approach reduced the training time by 96.68%.

## Figures and Tables

**Table 1:** Charging Conditions and Ratio of Used Data.

	Constant Current (A)	Upper Voltage Limit (V)	Cut-Off Current (mA)	Total Data Amount
#29	1.5	4.2	20	104,136
#30	1.5	4.2	20	104,136
#31	1.5	4.2	20	104,136
#32	1.5	4.2	20	104,136

**Table 2:** Error Evaluation Results.

	Errors	MSE	MAPE	Used Data Rate	Training Time
Data					
	40 × 80	2.41 × 10^−4^	1.28%	100.00%	1 min 38 s
First columns	40 × 2	5.10 × 10^−4^	1.87%	2.50%	6 s
	40 × 4	2.46 × 10^−4^	1.36%	5.00%	4 s
	40 × 6	2.41 × 10^−4^	1.33%	7.50%	7 s
	40× 8	2.50 × 10^−4^	1.34%	10.00%	24 s
PC	40 × 8	3.20 × 10^−4^	1.54%	10.00%	5 s
	40 × 20	5.02 × 10^−4^	2.01%	25.00%	60 s
Fusion data	40 × 4	2.39 × 10^−4^	0.89%	5.00%	4 s

**Table 3:** Charging Conditions and Amount of Data Used.

	Constant Current (mA)	Upper Voltage Limit	Cut-off Current (mA)	Total Data Amount
Battery #1	20	4.2	2	1,238,394
Battery #2	20	4.2	2	1,094,865
Battery #3	20	4.2	2	1,230,828
Battery #4	20	4.2	2	1,071,722

**Table 4:** Error evaluation results.

	Errors	MSE	MAPE	Used Data Rate	Training Time
Data Type					
Original	30 × 60	2.14 × 10^−4^	1.20%	100.00%	2891 s
First columns	30 × 2	4.40 × 10^−4^	1.75%	3.30%	295 s
	30 × 4	2.36 × 10^−4^	1.28%	6.70%	203 s
PC	30 × 4	3.39 × 10^−4^	1.47%	6.70%	98 s
	30 × 14	5.35 × 10^−4^	1.69%	22.10%	68 s
Fusion data	30 × 4	2.09 × 10^−4^	0.96%	6.70%	96 s

## Data Availability

The NASA’s batteries data can be found at https://ti.arc.nasa.gov/tech/dash/groups/pcoe/prognostic-data-repository/ (accessed on 1 December 2021). The rest data used to support the findings of this study are available from the corresponding author upon request.

## Nomenclature

$b_{f},\;\;b_{i}$, $b_{c}$, $b_{o}$	The bias of different processing units in LSTM
$w_{f},\;\;w_{i}$, $w_{c}$, $w_{o}$	The weights of different processing units in LSTM
σ	Sigma function
μ	The mean value of input data
${\tilde{x}}_{i}$	The normalized value of input data
$v^{\prime },\;\Delta v^{\prime }$ $b_{j}$	The normalized voltage value and voltage derivative value The principal component contribution rate
$a_{p}$	Principal components accumulative contribution rate
$PC_{i}$	Principal components matrix
